# Impact of a Healthy Weight Intervention Embedded Within a National Home Visiting Program on the Home Food Environment

**DOI:** 10.3389/fpubh.2018.00178

**Published:** 2018-06-26

**Authors:** Rachel G. Tabak, Alexandra B. Morshed, Cynthia D. Schwarz, Debra Haire-Joshu

**Affiliations:** ^1^The Brown School, Washington University in St. Louis, St. Louis, MO, United States; ^2^The Prevention Research Center, Washington University in St. Louis, St. Louis, MO, United States

**Keywords:** home environment, food availability, eating distractions, obesity intervention, research translation

## Abstract

**Purpose:** To determine whether a lifestyle intervention embedded within Parents as Teachers (PAT), a national child development and parenting home visiting program, helped families make food-related home environment changes.

**Design:** Secondary data analysis of a stratified randomized pragmatic trial. (Trial Registration: This study is registered at www.clinicaltrials.gov NCT01567033).

**Setting:** Participant homes in St. Louis, Missouri.

**Subjects:** Women (*n* = 179 with pre-post data, of 230 with baseline) participating in standard PAT, with overweight or obesity, and at least one preschool child with BMI percentile ≥60%.

**Intervention:** PAT + Healthy Eating and Active Living Taught at Home (HEALTH), embedded elements of the Diabetes Prevention Program within the standard PAT curriculum. PAT + HEALTH addressed specific behaviors that impact caloric intake (e.g., sugar-sweetened beverages), focusing on behavioral and environmental strategies. Consistent with PAT practice, the frequency, number, and focus (i.e., time spent on intervention components) of home visits were determined by the family's needs; dose structure was flexible [on average intervention: 23 (SD = 9), usual care: 13 (SD = 6) visits].

**Measures:** Food availability/accessibility and distractions in the home were assessed with items drawn largely from the HomeSTEAD Survey.

**Analysis:** Generalized estimating equations (GEEs) were used to test equality of changes between baseline and 24 months in the intervention and usual care groups.

**Results:** The only significant difference in the pattern of change between usual care and intervention was soda availability/accessibility (*p* = 0.013).

**Conclusion:** This embedded intervention successfully reduced availability/accessibility of sugar-sweetened beverages in the home. However, given the limited impact on other food-related home environment factors, future interventions could seek to more effectively intervene on all aspects of the home environment.

## Introduction

Obesity is prevalent in the United States and worldwide (1, 2), and is associated with diabetes and other acute and chronic conditions (2). Healthy eating behaviors can help reduce obesity and the risk of associated negative conditions (3). It is important therefore to develop strategies that are easy to communicate to individuals and to address modifiable factors, which can be delivered with broad reach through organizations with existing intervention delivery infrastructure.

Research on children and youth has indicated that the home environment and meal practices have important associations with dietary behaviors (4–10). This includes negative associations between availability of unhealthy food choices, and intake of these foods (8, 9, 11–13). For example, a review by Verloigne et al. found a positive relationship between consumption of soft drinks and availability of these beverages at home (12). While this research in adults has been limited, some studies have found associations between food available in the home and weight and dietary behaviors, particularly for women (14–16). For example Wansink et al. (17), found that normal weight women were more likely to store soft drinks out of sight than obese women (17). However, this research has primarily drawn from observational studies, making it difficult to determine the direction of the associations.

Further studies have explored the associations between distraction during meals, such as TV and other technology, unhealthy eating behaviors (18–23), and obesity (24, 25). Most research has been in children and adolescents (19, 20, 22, 23), and has focused on associations with eating behaviors rather than obesity. While associations with weight status have been mixed (24–27), reducing the number of meals eaten with the TV on has been suggested as an important intervention target (28).

Based on the evidence of associations and the role of the home environment in influencing behavior in ecological models (29), home environment modifications to facilitate healthy habits could be intervention targets (14). Examples of such modifications include storing sugar-sweetened beverages so they are out of site and difficult to access. To inform intervention efforts, this study aimed to determine whether an intervention targeting the home environment was successful in helping families make food-related home environment changes.

## Material and methods

### Design

The data for this study are drawn from a pragmatic trial promoting lifestyle change to prevent weight gain in women (30), implemented within Parents as Teachers (PAT) affiliated programs located across eight St. Louis, Missouri regions (31), which has been described elsewhere (32). PAT is a national home visiting program that promotes school readiness through optimal early development, learning, and health in young children by supporting and engaging their parents and caregivers. The program is delivered by trained parent educators and is offered free of charge to families. Stratified random sampling was used to select PAT participants within each region; the number of participants selected per region was proportional to region size.

Data were collected at baseline, 12- and 24-month follow-up. Participants received a $50 gift card for completing the measures at each time point. This is a secondary analysis, which was not included in the original aims and hypotheses of the study. The Washington University in St. Louis Institutional Review Board approved the study protocol and all participants provided written informed consent (Trial Registration: This study is registered at www.clinicaltrials.gov NCT01567033). Additional details about the study methods, including the CONSORT diagram are provided elsewhere (32).

### Sample

Participants were recruited on a rolling basis from April 2012 to September 2014. Study staff screened participants for eligibility and obtained consent on site or called mothers interested in participating. Inclusion criteria aimed to mirror real world PAT practice, thus the sample included female participants, 18–45 years of age, with a BMI 25–45 kg/m2, with at least one preschool child at risk for overweight (BMI percentile ≥60%) living in the home, who planned to continue in the PAT program for 2 years, and who were able to give informed consent. Women were excluded if they were currently pregnant or planned to become pregnant in the next 24 months, unable to speak English, currently enrolled in a weight loss program, undergoing treatment for diabetes or eating disorders, or unable to engage in a walking program.

### Intervention groups

Participants in usual care received the standard, evidence-based home visiting PAT program for parents of preschoolers that uses a strength-based, solution-focused model (33). Intervention participants received PAT + Healthy Eating and Active Living Taught at Home (HEALTH) [(32) has additional detail including a full description of the lesson topics and objectives 32], which adapted and embedded the Diabetes Prevention Program lifestyle intervention (34) within the standard PAT curriculum. Incorporation of healthy weight content into the curriculum was guided by Social Cognitive Theory and focused on core behavior change constructs addressing intrapersonal influences (e.g., constructs of self-assessment, reinforcement), interpersonal influences (e.g., observational learning/parental modeling), and influences of the home environment (e.g., food access, TV with meals) (35). Based on formative work, the intervention was simplified to address very specific high-risk behaviors that impact the caloric intake of mothers of young children with significant time constraints. This included a focus on behavioral and environmental strategies such as limiting intake of sugar sweetened beverages. Other behavioral strategies included substituting fruits and vegetables for high calorie snacks, limiting portion sizes, increasing physical activity, and decreasing sedentary activity. An important component for all of these behavioral targets were strategies to modify the home environment to facilitate healthy choices, such as storing unhealthy food items out of sight and out of reach. Consistent with PAT practice, the frequency and number of visits were determined by the family's needs and preferences as was the focus and time spent on each intervention component, therefore the dose structure for the intervention is flexible.

Parent educators with PAT experience and certified on the usual care curriculum [*N* = 9; mean age 44 years (range 29–67); 67% with a college degree] received additional training on the intervention (e.g., importance of parental lifestyle and modeling on child weight and development). Consistent with PAT training protocols, training took place over 1 day (8 h) and was in person. Fidelity monitoring, was conducted through lesson plan checklists on which parent educators documented content delivery and audiotaped home visits, which were reviewed by expert study staff as well as two randomly observed home visits a year for each parent educator (32).

### Data collection and measurement

To understand the impact of the intervention beyond the primary outcome (maternal weight; results published elsewhere) (32), surveys were also administered. Measures drawn largely from the HomeSTEAD Survey were used to assess home environment characteristics hypothesized to relate to healthy weight behaviors that were targets of the intervention. Based on these hypotheses, items that mapped on to two scales [i.e., Food availability and accessibility (2 items) and Distractions (6 items)] in the framework informing the HomeSTEAD measure, both within the “Structure” category, were included (22, 23). Food availability and accessibility items asked parents to report whether the soda and snacks in their home were “Easily accessible and in plain sight”; “Accessible but out of sight;” or “Hidden and out of reach.” The distraction items asked about the frequency the TV was on at breakfast, lunch, and dinner, the frequency the parent eats meals and snacks with their child while watching TV, and the frequency of technology (e.g., cell phone) use during family meals. Full item wording is available in the Supplementary Table1. Research staff measured participants' height and weight in accordance with NHANES procedures (36). Sociodemographic measures (such as: age, marital status, number of children, and monthly income) were assessed by survey.

### Analysis

Baseline characteristics were compared for the intervention and usual care participants included in the analysis (i.e., those with data at 12 and/or 24 months; repeated measures analysis allows for inclusion of data from participants reporting at only 12 months, only 24 months, or both 12 and 24 months) by unpaired *t*-test (for normally distributed continuous variables), Wilcoxon's test (for ordinal variables), or chi-square test (for binary variables).

Generalized estimating equations (GEEs) using a binomial distribution, logit link function, and exchangeable correlation structure (binary outcomes) or multinomial probability distribution, cumulative logit link function, and independent correlation structure (ordinal outcomes) were employed to test hypotheses regarding equality of changes over time, and included: treatment group, timepoint, and group by timepoint interaction. For outcomes with a significant interaction term, which was this study's main statistic of interest and would indicate a difference in change over time between groups, statistical contrasts within the GEE were used. These contrasts tested null hypothesis that changes between two timepoints in the usual care group were equal to corresponding changes in the intervention group. Analyses were conducted using the Statistical Analysis Systems (SAS) version 9.4 (SAS Institute, Care, NC, USA).

## Results

Of the 230 participants who completed the baseline assessment, 179 (78%) completed a 12- and/or 24-month follow-up assessment. One hundred seventy-three (75%) participants completed a 12-month follow-up, and 156 (68%) completed a follow-up at 24 months. Most of the women in the sample were currently married (61%). The mean age of the racially/ethnically (59% white, 32% African American or black, 7% other), and economically diverse (51% receiving WIC or other program assistance) participants who completed the study was 32 (SD 6) years. As shown in Table 1, most participants (63%) had class I or class II obesity, while 20% were overweight and 18% had class III obesity. There were no significant differences between usual care or intervention participants at baseline, with one exception: intervention participants were generally less educated than participants in the usual care group (Table 1). On average, participants in the intervention group completed 23 (SD 9) home visits over 24 months, and those in the usual care group completed 13 (SD 6); the average length of visits for both groups was 63 min (SD 11).

**Table 1:** Baseline characteristics for participants enrolled in the usual care and intervention groups of the intervention embedded in parents as teachers, who provided follow-up data at any timepoint (12 and/or 24 months).

	**Total cohort (No. = 179)**	**By treatment group**	
		**Usual care (No. = 97)**	**Intervention (No. = 82)**
**VARIABLES**			
Age (year), mean (SD)	32 (6)	33 (5)	32 (6)
**RACE, NO. (%)**			
Black or African American	57 (32%)	30 (31%)	27 (33%)
White	105 (59%)	59 (61%)	46 (56%)
Other	13 (7%)	8 (8%)	5 (6%)
Unknown, not reporting race	4 (2%)	0 (0%)	4 (5%)
**HIGHEST GRADE OF SCHOOL COMPLETED, NO. (%)**			
Some high school, high school graduate	25 (14%)	10(10%)	15(19%)
Some college/technical/vocational school	65 (37%)	33 (34%)	32 (40%)
College or university graduate	56 (31%)	34 (35%)	22 (27%)
Graduate or professional school	32 (18%)	20 (21%)	12 (15%)
Presently married, No. (%)	109 (61%)	59 (61%)	50 (61%)
**ANNUAL HOUSEHOLD INCOME FROM ALL SOURCES, NO. (%)**			
Under $30,000	66 (39%)	36 (38%)	30 (40%)
$30,000–$74,999	62 (36%)	34(36%)	28(37%)
$75,000- Over $100,000	42(25%)	25 (26%)	17 (23%)
WIC or other program assistance, No. (%)	89 (51%)	43 (46%)	46 (58%)
Weight (kg), mean (SD)	93 (15)	93 (15)	93 (16)
BMI (kg/m^2^), mean (SD)	34.4 (5.2)	34.5 (5.2)	34.4 (5.3)
**OBESITY CATEGORY, NO. (%)**			
Overweight (BMI 25.0–29.9)	35 (20%)	19 (20%)	16 (20%)
Obese I (BMI 30.0–34.9)	68 (38%)	35 (36%)	33 (40%)
Obese II (BMI 35.0–39.9)	44 (25%)	28 (29%)	16 (20%)
Obese III (BMI ≥25.0)	32 (18%)	15 (15%)	17 (21%)

*SD, standard deviation; No. , number of participants; %, percent of treatment group; IQR, interquartile range defined as the 75th minus the 25th percentile; n/a, not applicable. Due to missing data, sample sizes may vary across variables*.

Table 2 indicates that the home environment factor for which the interaction between intervention group and time was significant was availability of soda in the home. This indicates there was a significant difference in the pattern of change in soda availability between the usual care and intervention groups. The contrast analysis indicated that the change from baseline to 12 months (*p* = 0.013) and the change from baseline to 24 months (*p* = 0.003) were significantly different between the usual care and intervention groups, but the change from 12 to 24 months did not differ significantly (*p* = 0.423). At baseline, 28% of participants in the usual care group indicated soda in their home was easily accessible and in plain sight, 50% indicated soda was accessible but out of sight, and 23% indicated the soda in their home was hidden and out of reach. Soda availability in this group changed little over the two time periods. However, in the intervention group, 35, 42, and 23% of participants reported soda in their home was easily accessible and in plain sight, accessible but out of sight, and hidden and out of reach, respectively, at baseline; this had improved to 13, 36, and 52% at 12 months, and to 14, 44, and 42% at 24 months.

**Table 2:** Home environment outcomes at baseline, 12 and 24 months and Test^a^ for differences in trajectory over these timepoints for participants in the usual care and intervention groups of the HEALTH intervention embedded in parents as teachers.

**Outcome measures**		**Baseline *n* (%)**	**12 month *n* (%)**	**24 month *n* (%)**	***p*-value^b^**
**DISTRACTION**					
TV on at Breakfast?	Usual Care				
	Never/Rarely	65 (67.7)	65 (69.9)	63 (75.0)	
	Sometimes/Often/Always	31 (32.3)	28 (30.1)	21 (25.0)	
	Intervention				
	Never/Rarely	57 (71.3)	59 (77.6)	54 (77.1)	
	Sometimes/Often/Always	23 (28.8)	17 (22.4)	16 (22.9)	0.748
TV on at Lunch?	Usual Care				
	Never/Rarely	37 (50.0)	39 (55.7)	44 (63.8)	
	Sometimes/Often/Always	37 (50.0)	31 (44.3)	25 (36.2)	
	Intervention				
	Never/Rarely	36 (64.3)	42 (77.8)	34 (69.4)	
	Sometimes/Often/Always	20 (35.7)	12 (22.2)	15 (30.6)	0.203
TV on at Dinner?	Usual Care				
	Never/Rarely	36 (37.5)	40 (43.5)	45 (53.6)	
	Sometimes/Often/Always	60 (62.5)	52 (56.5)	39 (46.4)	
	Intervention				
	Never/Rarely	36 (45.0)	38 (50.0)	35 (50.0)	
	Sometimes/Often/Always	44 (55.0)	38 (50.0)	35 (50.0)	0.235
Eat dinner in front of the TV with your child?	Usual Care				
	Never/Rarely	50 (52.1)	60 (64.5)	54 (65.1)	
	Sometimes/Often/Always	46 (47.9)	33 (35.5)	29 (34.9)	
	Intervention				
	Never/Rarely	50 (63.3)	49 (64.5)	52 (74.3)	
	Sometimes/Often/Always	29 (36.7)	27 (35.5)	18 (25.7)	0.313
Eat snacks with your child while watching TV?	Usual Care				
	Never/Rarely	30 (31.3)	32 (34.4)	24 (28.6)	
	Sometimes/Often/Always	66 (68.8)	61 (65.6)	60 (71.4)	
	Intervention				
	Never/Rarely	30 (38.0)	36 (47.4)	39 (55.7)	
	Sometimes/Often/Always	49 (62.0)	40 (52.6)	31 (44.3)	0.074
Cell phones, etc. during family meals?	Usual Care				
	Never/Rarely	61 (64.2)	60 (65.9)	56 (68.3)	
	Sometimes/Often/Always	34 (35.8)	31 (34.1)	26 (31.7)	
	Intervention				
	Never/Rarely	52 (65.0)	52 (69.3)	50 (71.4)	
	Sometimes/Often/Always	28 (35.0)	23 (30.7)	20 (28.6)	0.938
**FOOD AVAILABILITY AND ACCESSIBILITY**					
I have soda at home that is:	Usual Care				
	Easily accessible and in plain sight	22 (27.5)	22 (27.9)	18 (25.7)	
	Accessible but out of sight	40 (50.0)	33 (41.8)	33 (47.1)	
	Hidden and out of reach	18 (22.5)	24 (30.4)	19 (27.1)	
	Intervention				
	Easily accessible and in plain sight	23 (34.9)	8 (12.9)	8 (13.6)	
	Accessible but out of sight	28 (42.4)	22 (35.5)	26 (44.1)	
	Hidden and out of reach	15 (22.7)	32 (51.6)	25 (42.4)	**0.013**
I have sweet or salty snack foods at home that is:	Usual Care				
	Easily accessible and in plain sight	25 (26.3)	21 (23.3)	19 (23.5)	
	Accessible but out of sight	53 (55.8)	40 (44.4)	41 (50.6)	
	Hidden and out of reach	17 (17.9)	29 (32.2)	21 (25.9)	
	Intervention				
	Easily accessible and in plain sight	23 (29.5)	12 (16.7)	12 (17.9)	
	Accessible but out of sight	42 (53.9)	43 (59.7)	35 (52.2)	
	Hidden and out of reach	13 (16.7)	17 (23.6)	20 (29.9)	0.542

*n, number of participants; %, percent of home environment characteristic category. ^a^GEE models included main effects: treatment group and timepoint, and the interaction between treatment group and timepoint. ^b^p-values presented are for the interaction term in the GEE model. Bold values indicates p < 0.05*.

Among the distraction items, there were no significant differences between the usual care and intervention groups in the pattern of change over the study period.

## Discussion

Our findings demonstrate the impact of the HEALTH intervention on an important home environment target. Intervention group participants created a home environment with less easily accessible soda over 12 and 24 months of follow-up, while soda access in the homes of usual care participants did not change. This is particularly important given the previously documented benefit of the intervention on intake of added sugars and added sugars from sugar sweetened beverages as well as body weight and body mass index (32). However, HEALTH's impact was observed for only one of the home environment measures targeted.

Availability and accessibility of soda in the home was the only home environment factor impacted by HEALTH, which may be because reducing sugar-sweetened beverage intake was an important focus of the intervention; this is also a specific, measureable behavior, which may have been easier for parent educators to promote with their families (32). Embedding HEALTH within the existing, reimbursable home visits provided by PAT facilitates changes to the home environment, as parent educators work with a family in their home. This gives the educator an understanding of the family's context and can help the mother make changes to her home environment to facilitate dietary behavior changes. One such change is encouraging the mother to remove cues to drink sugar-sweetened beverages such as storage of the beverage in a place that is easily accessible or soda that is in plain sight.

Another advantage to embedding HEALTH within PAT is the ability to interact with the family over a number of visits for a sustained period of time. While 23 visits over 2 years may seem intensive, this is only 10 additional visits over 2 years more than the 13 visits usual care PAT families received. The 23 visits fit within PAT's reimbursable visit structure, which allows for additional visits for families with at least two high needs characteristics (e.g., low educational attainment, low income, parent or child with disabilities/chronic health condition, recent immigrant family, parent with mental illness, and unstable housing) (37). This allows the visit structure and content to be tailored to the family's needs, which may require additional contact to solidify behavior change over time.

This study also informs interventions targeting other environmental changes associated with distractions (e.g., TV and eating) (38). Several studies have documented the relationship between TV and caloric intake or obesity (39–41), with some studies suggesting the impact of parents limiting TV exposure in young children (38) and removal of TVs from child bedrooms (42). However there is limited evidence of the impact of strategies to reduce family TV watching (43), particularly during mealtimes (38). One study of a home environment intervention found improvements in meals eaten in front of the TV as well as number of servings of salty/fatty snacks available in the home; this study was not limited to parents with overweight and obesity [mean (standard deviation) BMI at baseline: intervention: 29.0 (7.43); control: 28.98 (5.40)], suggesting there may be differences in responsiveness to interventions based on initial weight status (44). Our data suggest interventions requiring TV limits for multiple family members do not respond to interventions primarily targeting the primary caregiver. Additional research may be needed to identify and test more comprehensive family based strategies to promote environmental change and impact.

Other intervention studies have targeted changes to the home environment as a way to promote behavior change (14, 45). Gorin et al. (14) found that an intervention which included a behavioral component as well as a home environment focused intervention had benefits on the home environment as well as a stronger impact on weight loss at 6 months compared with a group that received only the behavioral component. Differences in the food environment and weight were no longer present at the 18 month follow up in the total sample, but the weight outcomes were persistent among women (15). While only one home environment factor was significantly impacted by the intervention, qualitative research has indicated even small changes can be challenging to implement (46). Further, other intervention trials have been efficacious in terms of modifying some home environment characteristics but not others (44, 45).

This study has limitations worth noting. Home environment data were collected by self-report, creating the possibility for social desirability bias. However, the only significant change observed in the home environment was availability of soda; if parents were reporting socially desirable answers, it might be anticipated that changes to numerous home environment characteristics would be observed. In addition, there were significant differences in objectively assessed weight between the intervention and control groups over 24 months (32). Also, while the items included mapped on to the framework informing the HomeSTEAD measure (22, 23), not all items from each selected scale were included. Additionally, several items that are not part of the HomSTEAD measure were included, as the HomeSTEAD tool does not include the relevant items. This may reduce the reliability and validity evidence for the environmental assessment tool. This study also included multiple comparisons, increasing the risk for Type 1 error, and in that it is an exploratory, secondary analysis, such that the original trial was not powered to detect differences in these outcomes. Finally, the study sample has limitations regarding sample size (*n* = 179) and generalizability as the population were all families from St. Louis, Missouri.

## Conclusion

It is possible to embed an intervention to promote a healthy home environment within the reimbursable visit structure of a national home visiting program. Using targeted, specific strategies, these visits can help parents make food-related home environment changes to facilitate healthy choices. However, given the limited impact on other food-related home environment factors, future interventions could seek to more effectively intervene on all aspects of the home environment. Interventions to promote healthy eating behaviors and prevent obesity should target the home environment to reduce availability and accessibility of sugar-sweetened beverages, a relatively low cost, easily modifiable factor.

## Data availability statement

The datasets for this manuscript are not publicly available because of confidentiality requirements. Requests to access the datasets should be directed to Rachel Tabak (rtabak@wustl.edu).

## Author contributions

RT and AM analyzed the data. DH-J and CS designed and managed the study. All authors (RT, AM, CS, and DH-J) contributed to interpretation of results and prepared the manuscript.

### Conflict of interest statement

The authors declare that the research was conducted in the absence of any commercial or financial relationships that could be construed as a potential conflict of interest.
