# Supplementary material for: Impact of a Healthy Weight Intervention Embedded Within a National Home Visiting Program on the Home Food Environment
Source: Front Public Health. 2018 Jun 26;6:178. doi: 10.3389/fpubh.2018.00178 (PMC6028746; doi:10.3389/fpubh.2018.00178)
Supplement: Supplementary file 1 [file Data_Sheet_1.pdf]

Supplementary Table. Full survey item wording

Distraction

During a typical day, how often is the TV on during you meals, even if you aren't watching it?

a. Breakfast
 Never
 Rarely
 Sometimes
 Often
 Always

b. Lunch
 1 Never
 2 Rarely
 3 Sometimes
 4 Often
 5 Always

c. Dinner
 Never
 Rarely
 Sometimes
 Often
 Always

How often do you and/or another adult in the household. . .

a. Eat dinner in front of the TV together with your child
 Never
 Rarely
 Sometimes
 Often
 Always

b. Eat snacks together with your child while watching TV
 Never
 Rarely
 Sometimes
 Often
 Always

How often do members of your family (parents or children) use cell phones, iPods, laptops or video games during family meals?
 Never
 Rarely
 Sometimes
 Often
 Always

Food availability and accessibility

I have soda at home that is:
 Easily accessible and in plain sight

Accessible but out of sight
Hidden and out of reach

I have sweet or salty snack foods that are:
Easily accessible and in plain sight
Accessible but out of sight
Hidden and out of reach
